# Assessment of Potential Toxic Effects of RNAi-Based Transgenic Cotton on the Non-Target Predator *Harmonia axyridis*

**DOI:** 10.3390/biology14091173

**Published:** 2025-09-02

**Authors:** Haiqin Yao, Haonan Xu, Jun Yang, Weihua Ma

**Affiliations:** 1 College of Food Science and Technology, Wuhan Business University, Wuhan 430056, China; yaohq@wbu.edu.cn; 2 Hubei Insect Resources Utilization and Sustainable Pest Management Key Laboratory, College of Plant Science and Technology, Huazhong Agricultural University, Wuhan 430070, China

**Keywords:** risks assessment, RNAi, *Adelphocoris suturalis*, plant–pest–natural enemy, ds*AsFAR*

## Abstract

Although traditional genetically modified (GM) cotton has reduced lepidopteran pests, secondary pests such as *Adelphocoris suturalis* Jakovlev (Hemiptera: Miridae) have become increasingly problematic. RNA interference (RNAi)-based insect-resistant plants offer a promising alternative, but their potential ecological risks, particularly within the plant–pest–natural enemy framework, require comprehensive evaluation. As a key natural enemy in cotton agroecosystems, *Harmonia axyridis* Pallas (Coleoptera: Coccinellidae) plays a vital role in regulating pest populations and thus serves as an important non-target insect for environmental risk assessment. In this study, ds*AsFAR* transgenic cotton and the non-target insect *H. axyridis* were used to evaluate potential ecological risks. Based on the sequences of the *HaFAR*, *AsFAR*, and *GFP* genes, ds*AsFAR*, ds*HaFAR*, and ds*GFP* were synthesized in vitro and incorporated into artificial diets fed to *H. axyridis*, along with a ddH_2_O control group. No significant differences were observed among the treatments across various developmental stages. Furthermore, *H. axyridis* was fed aphids that had been reared on either transgenic or non-transgenic cotton plants cultivated in a greenhouse. The findings indicated no significant differences in the growth, development, predatory ability, or fecundity of *H. axyridis*. These results suggest that transgenic ds*AsFAR* cotton targeting *A. suturalis* poses no detectable adverse effects on *H. axyridis*, supporting the environmental safety of RNAi crops within a plant–pest–natural enemy ecological interaction framework. Moreover, the ds*AsFAR* expressed in transgenic cotton was not effectively transferred through the food chain to affect homologous gene expression in *H. axyridis*.

## Simple Summary

The deployment of genetically engineered (GE) crops, particularly RNA interference (RNAi) crops that express double-stranded RNA, has raised biosafety concerns among regulatory authorities, the scientific community, and the public. Such crops may present potential risks to public health, agricultural sustainability, and biodiversity. Natural enemies of insect pests, including the lady beetle *Harmonia axyridis*, play a crucial role in pest regulation and maintaining ecosystem balance. This study assessed the potential effects of RNAi cotton targeting the pest *Adelphocoris suturalis* on *H. axyridis*. Both laboratory and field experiments demonstrated no significant adverse impacts on the beetle’s growth, development, predatory efficacy, or reproductive capacity. Furthermore, the RNAi construct was not effectively transmitted through the food chain. These results support the environmental safety of RNAi cotton within the plant–pest–natural enemy ecological framework. This contributes to the advancement of sustainable agroecosystems, the conservation of biodiversity, and the production of safer food supplies, ultimately benefiting agriculture and society by enhancing crop protection while minimizing ecological risks.

## 1. Introduction

RNA interference (RNAi) is an evolutionarily conserved post-transcriptional gene silencing (PTGS) phenomenon in which the introduction of dsRNA leads to the degradation of specific mRNA and inhibits the normal expression of target genes [1,2]. Matzke was the first to report the phenomenon of gene silencing caused by tobacco co-transfection [3]. With the rapid development of modern molecular technology, RNAi is increasingly being recognized as one of the leading approaches for managing major agricultural pests due to its environmentally friendly, efficient, and target-specific characteristics [4]. The quality of various crops has been improved by using RNAi technology, for example, via interfering with the function of fatty acid dehydrogenase, which significantly increases the proportion of oleic acid in rapeseed [5]. Including RNAi crops, insect-resistant genetically engineered (IRGE) crops that use RNAi have been released to the market [6]. In order to gain a deeper understanding of their inherent risks to the ecological environment, most countries that have approved the environmental release of RNAi crops have begun to develop regulatory frameworks to evaluate the potential hazards of using this new biotechnology [7,8,9]. However, there has been no standard framework for assessing the environmental risk of RNAi crops in China up to now.

Cotton is widely cultivated as a cash crop worldwide, but its safe production is threatened by various factors, such as agricultural pests, diseases, drought, soil salinization, and so on [10,11]. In view of the above reasons, genetically modified technology has profound implications for cotton cultivation. With the continuous development of molecular biotechnology, the planting area of transgenic cotton has reached more than 90% in China [12]. The popularization and cultivation of traditional genetically modified (GM) insect-resistant cotton has significantly reduced the population density of lepidopteran pests such as cotton bollworms in the field. However, due to large-scale monoculture and a reduction in the use of chemical pesticides, minor pests such as mites and aphids have gradually become rampant and become major pests. Among them, *A. suturalis* and *Apolygus lucorum* Meyer—Dür (Heteroptera: Miridae) can cause serious damage to cotton production and quality. Transgenic ds*AsFAR* cotton can inhibit the fecundity of *A*. *suturalis* by silencing its *FAR* gene expression. This type of dsRNA transgenic cotton can produce significant control effects on field populations of *A. suturalis* and has great application prospects in production. However, its ecological safety within the plant–pest–natural enemy framework has not yet been fully evaluated. *H. axyridis*, a common natural enemy insect, plays a crucial ecological role in cotton field ecosystems by preying on various pests. As a representative species in the plant–pest–natural enemy trophic system, *H. axyridis* is ecologically significant for maintaining pest population balance. Moreover, as a member of the Coleoptera order, *H. axyridis* has been reported to exhibit higher sensitivity to dsRNA compared to insects from other insect orders, making it an ideal test organism for evaluating the biosafety of transgenic dsRNA cotton in non-target beneficial insects [13].

A previous study conducted by our team found that the direct feeding of ds*AsFAR* to *H. axyridis* had no significant impact on their own growth, development, reproduction, or other physiological processes, and that GM cotton does not affect physiological indicators of *H. axyridis* through the food chain [14]. However, functional genomics research shows that RNAi often induces off-target effects in cells or organisms, which is a major drawback of this technology [15,16,17]. The off-target effects of RNAi on non-target organisms may be sublethal, and such effects may not be readily detectable through biological assays alone. Additionally, organisms possess a vast number of genes, complicating the assessment of these effects. Omics analysis techniques [18] offer a comprehensive approach to detecting the off-target effects of RNAi transgenic crops by examining global transcriptome changes. We employed qRT-PCR to investigate the relationship between dsRNA uptake efficiency and the duration of feeding. Additionally, transcriptome sequencing technology combined with quantitative analysis verification was utilized to assess the off-target effect of dsRNA. Furthermore, to anticipate the potential impact of off-target effects from transgenic dsRNA crops, this study employed RNAi technology to predict the possible consequences for non-target organisms. This approach provides a useful reference for evaluating the safety of non-target organisms consuming transgenic dsRNA crops over multiple generations under field conditions.

## 2. Materials and Methods

### 2.1. General Procedures

#### 2.1.1. Insect Rearing

The initial population of *H*. *axyridis* was kindly provided by Xingmiao Zhou (Huazhong Agricultural University, Wuhan, China). The beetles were reared for several generations in an artificial climate chamber maintained at 25 ± 2 °C, 65 ± 5% relative humidity, and a 16 h light/8 h dark photoperiod. The insects were fed *Acyrthosiphon pisum* Harris (Hemiptera: Aphididae) reared on *Vicia faba* L. (Fabales: Fabaceae). To minimize cannibalism, larvae were placed individually in 90 mm Petri dishes containing moistened tissue paper. Artificial diet was provided daily, and larval development was monitored until adult emergence. The artificial diet was prepared as follows: (1) A dry mixture was made from pig liver powder, yeast, sucrose, linseed oil, olive oil, and β-carotene, thoroughly stirred with distilled water. (2) In parallel, agar and honey were dissolved in distilled water with heating. (3) The heated solution was boiled several times and added to the first mixture, then stirred to homogeneity; (4) The final mixture was dispensed into 24-well plates and stored at 4 °C after cooling below 50 °C.

#### 2.1.2. Cotton Cultivation

Seeds of transgenic ds*AsFAR* cotton and its non-transgenic parental cultivar (*Gossypium hirsutum* cv. Jin668) were obtained from Prof. Shuangxia Jin (National Key Laboratory of Crop Genetic Improvement, Huazhong Agricultural University). The ds*AsFAR* line expresses a 432 bp double-stranded RNA targeting the *AsFAR* gene, which was introduced via Agrobacterium-mediated transformation using the pHellsgate4 RNAi vector. Both lines were planted in pesticide-free experimental plots (10.5 m × 4.5 m) at Huazhong Agricultural University.

### 2.2. Experimental Procedures

#### 2.2.1. Trophic Transfer Assay

This assay was designed to evaluate the effect of transgenic cotton on *H. axyridis* via a tritrophic pathway. At the four-leaf stage, *A. pisum* aphids were transferred onto transgenic or control cotton plants and reared for three generations to ensure sufficient dsRNA uptake. Second-instar *H. axyridis* larvae were then introduced into Petri dishes (Φ = 90 mm) containing a fresh cotton leaf and aphid larvae. Life-table parameters—including survival, developmental time, and fecundity—were recorded. Each treatment included three replicates with 20 larvae per replicate.

#### 2.2.2. Artificial Diet Exposure Assay

This direct RNAi assay aimed to determine the effect of orally delivered dsRNA on *H. axyridis*. Artificial diets were mixed with ds*AsFAR*, ds*HaFAR* (positive control), ds*GFP* (negative control), or ddH_2_O. Each dsRNA was provided at a concentration 10-fold higher than that expressed in cotton leaves (7 µg/g). One newly molted 4th instar larva (<24 h) was placed in a Petri dish containing the treated diet. The diet was refreshed daily. Upon adult emergence, 10 individuals (6 females and 4 males) were housed in a 12 cm diameter, 10 cm high cylinder with *A. pisum* and three to four branches of *Jasminum nudiflorum* Lindl. (Oleaceae: Jasminum) as an oviposition substrate. Life-history traits—such as adult longevity, fecundity, and survival—were monitored. Each treatment was conducted with three replicates of 20 individuals. To ensure the reliability and validity of the experimental data, and to minimize potential confounding factors, the mortality rate of the control groups (artificial diets mixed with ds*GFP* or ddH_2_O) were kept below 10%.

### 2.3. Synthesis of dsRNAs

Pairwise sequence alignment was carried out to obtain the *HaFAR* gene sequence most homologous to *AsFAR*, which is expressed in ds*AsFAR* transgenic cotton (49.55%) from our local *H. axyridis* transcriptome. Subsequently, ds*AsFAR*, ds*HaFAR*, and ds*GFP* [19] were amplified by PCR using primers containing the T7 promoter (Table S1). The dsRNAs were synthesized using a dsRNA Synthesis Kit (Thermo Fisher Scientific, CA, USA).

### 2.4. Quantitative Real-Time PCR (qRT-PCR)

Following the manufacturer’s instructions, total RNA was extracted with the RNAiso reagent (Takara, Kyoto, Japan). cDNA was prepared using a reverse transcription kit (Takara, Kyoto, Japan). The 10 μL qRT-PCR reaction mixture consisted of 5 µL of SYBR solution (Takara, Kyoto, Japan), 0.8 µL of specific primers (Table S1), 2.2 µL of double-distilled water, and 2 µL of cDNA. The reaction mixture was placed in a 96-well microplate. The qRT-PCR was performed on the Bio-Rad Detection iQ2 System (Bio-Rad, Hercules, CA, USA). The qRT-PCR program was 95 °C for 30 s, followed by 40 cycles of 95 °C for 5 s and 60 °C for 30 s. The *H. axyridis 18S* gene was used as an endogenous reference gene for the run. The 2^−ΔΔCt^ method was used to calculate the relative transcript levels of the corresponding genes.

### 2.5. Off-Target Effects and Transcriptome Analysis

The samples collected above were also used for RNA-Seq on an Illumina platform at MetWare (Wuhan, China). The raw reads were filtered, and the clean reads were de novo assembled into unigenes using Trinity. Transcript abundance was then estimated using RSEM. Differential expression analysis was performed using DESeq2. The screening of differentially expressed genes (DEGs) was based on log_2_ (fold change) and false discovery rate (FDR). Genes with log_2_ (fold change) > 1 and FDR < 0.05 were considered DEGs. Several randomly selected DEGs from each group were validated using qRT-PCR. The off-target effects of dsRNA were assessed by analyzing the correlation between the number of matched base pairs and their fold changes for each gene containing continuously matched base pairs, using Perl scripts. Furthermore, two additional sets of non-target genes were also considered. The first one was the homologous genes of the target genes, which were identified using BLASTN (E-value < 1 × 10^−10^). The second one was genes in the same pathway as the target genes. In addition, the transcriptome was evaluated using the Shannon entropy count, which was calculated using R script. Defining transcriptomic changes as Shannon entropy allowed transcriptome variation to be displayed as a separate metric [20]. The bioinformatic analysis was adopted from our previous study [21]. The enrichment analysis was performed based on the hypergeometric test. For the Kyoto Encyclopedia of Genes and Genomes (KEGG) analysis, the hypergeometric distribution test was performed at the pathway level; for Gene Ontology (GO), it was performed based on the GO term. Data visualization was performed using R (version 4.2.0).

### 2.6. Statistical Analysis

GraphPad Prism 10.1.2 (Dotmatics, Boston, MA, USA) was used for the statistical analysis of the data. Data from the biological assays were analyzed using one-way ANOVA followed by Tukey’s multiple comparison tests, and data produced by qRT-PCR were analyzed using Student’s *t*-test.

## 3. Results

### 3.1. Uptake Efficiency of dsRNA in H. axyridis

**Figure 1:**
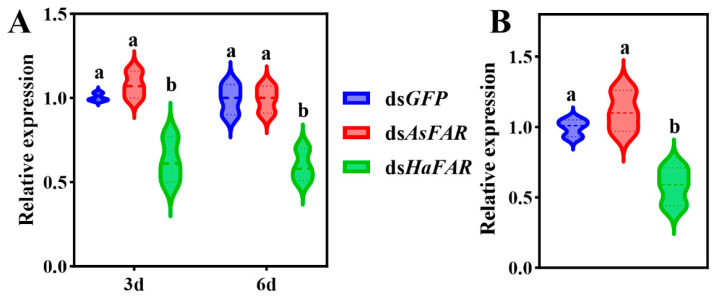
Changes in *FAR* gene expression in larvae and adults of *H. axyridis* after feeding on three dsRNAs. (**A**) Changes in *FAR* gene expression in larvae of *H. axyridis* after feeding on three dsRNAs for 3 d and 6 d (F_3d_ (2, 6) = 20.65, P_3d_ = 0.0020; F_6d_ (2, 6) = 19.83 P_6d_ = 0.0023). (**B**) Changes in *FAR* gene expression in adults of *H. axyridis* after feeding three dsRNAs for 3 d (F (2, 6) = 16.25, *p* = 0.0038). The values are represented as mean ± SD of three replicates. Error bars indicate the standard error of the mean. Different lowercase letters (a, b) indicated significant differences (*p*-value < 0.05). *p*-Values are based on multiple comparisons that were performed using one-way ANOVA followed by Tukey’s multiple comparison tests.

The expression level of dsRNA in ds*AsFAR* transgenic cotton is 0.7 μg/g. The dsRNA was added to the artificial diet, resulting in a final concentration of 7 μg/g (10-fold higher than in GM cotton) for ds*AsFAR*, ds*HaFAR* (positive control), and ds*GFP* (negative control), respectively. Both the larvae and adults of *H. axyridis* were fed on all dsRNAs for 3 and 6 days and 3 days, respectively, and then the changes in *HaFAR* gene expression levels in *H. axyridis* were detected after 24 h of feeding. The results showed that ingesting ds*AsFAR* caused no significant interference in the *HaFAR* expression in *H. axyridis* larvae and adults (Figure 1A,B). In addition, the dsRNA uptake efficiency did not change significantly when the larvae were fed on dsRNA for different lengths (3 d and 6 d) compared with the positive control (ds*HaFAR*). However, the positive control exhibited higher RNAi efficiency in *H. axyridis* adults.

### 3.2. Effect of Uptake dsRNA on the Life-Table Parameters of H. axyridis

**Figure 2:**
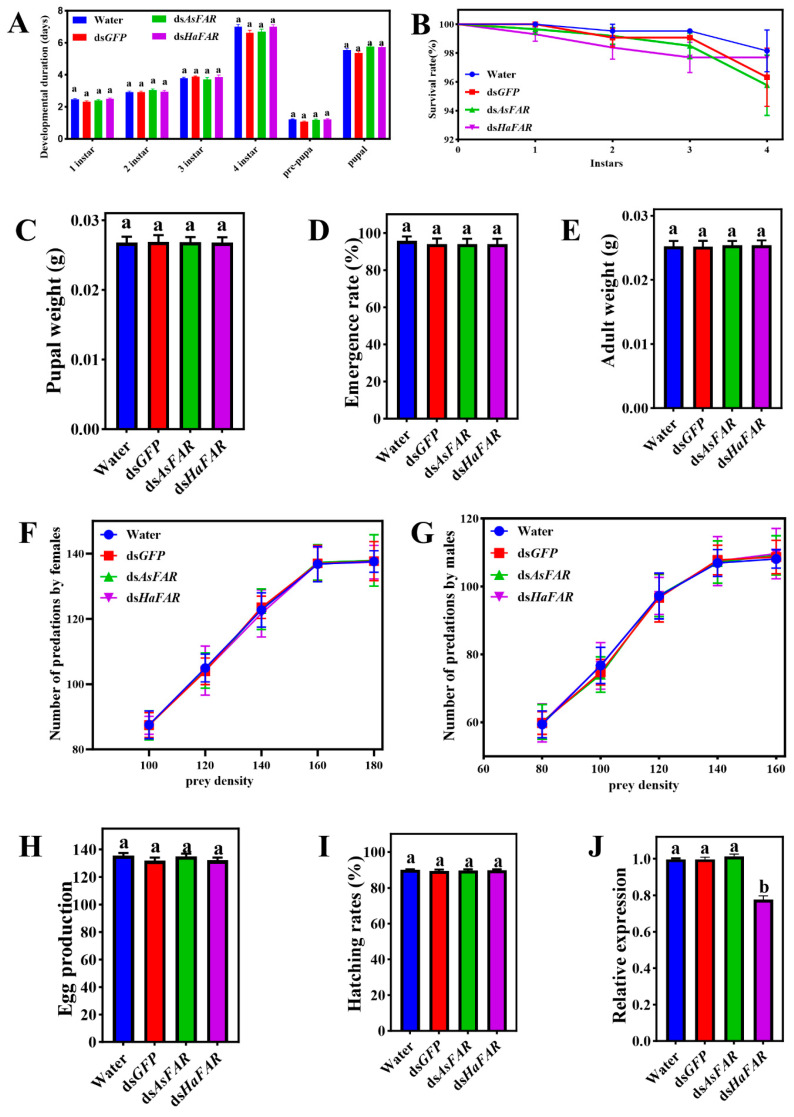
Evaluation of RNA interference of *FAR* in *H. axyridis*. Duration of the pre-imaginal stages (**A**), survival rates (**B**), pupal weight (F_1 instar_ (3, 282) = 1.85, P_1 instar_ = 0.14; F_2 instar_ (3, 278) = 0.6440, P_2 instar_ = 0.5873; F_3 instar_ (3, 276) = 0.67, P_4 instar_ = 0.5719; F_4 instar_ (3, 268) = 1.556, P_pre-pura_ = 0.2004; F_pre-pura_ (3, 268) = 1.533, P_pupal_ = 0.2063 F (3, 253) = 1.551, *p* = 0.2019) (**C**), emergence rate (F (3, 268) = 0.28, *p* = 0.84) (**D**), adult weight (F (3, 268) = 0.09, *p* = 0.97) (**E**), number of predations by females (**F**), number of predations by males (**G**), egg production (F (3, 56) = 0.77, *p* = 0.51) (**H**), and hatching rates (F (3, 268) = 0.28, *p* = 0.84) (**I**) for populations from all treatments. (**J**) Changes in *FAR* expression after feeding dsRNA-mixed artificial diets in *H. axyridis* (F (3, 8) = 209.14, *p* = 6.15 × 10^−8^). The values are represented as mean ± SD of three replicates. Error bars indicate the standard error of the mean. Different lowercase letters (a, b) indicate significant differences (*p*-value < 0.05). *p*-Values are based on multiple comparisons that were performed using one-way ANOVA followed by Tukey’s multiple comparison tests.

Even though a 10-fold (7 μg/g) high dosage was applied in the feeding test, no significant changes were observed in different developmental stages of *H. axyridis* that were separately treated with dsRNAs (ds*GFP*, ds*AsFAR,* and ds*HaFAR*) and H_2_O. The developmental durations of *H. axyridis* at each instar stage were relatively consistent (Figure 2A). All the survival rates of *H. axyridis* with dsRNA treatments were above 95%, but there was no significant difference among them (Figure 2B). In addition, the pupal weight (Figure 2C), emergence rate (Figure 2D), adult weight (Figure 2E), number of predations by females (Figure 2F), number of predations by males (Figure 2G), egg production (Figure 2H), and hatching rates (Figure 2I) for populations from all treatments showed no significant differences. Furthermore, the *HaFAR* gene expression level in the ds*HaFAR* treatment was significantly decreased compared with other treatments (*p*-value < 0.05, Figure 2J), indicating that *H. axyridis* can acquire exogenous dsRNA through feeding. Overall, these results demonstrate that ds*AsFAR* had no significant effect on the survival, development, predation ability, fecundity, or *HaFAR* expression of *H. axyridis* at any stage.

### 3.3. The Vital Activities of H. axyridis Were Not Affected by the Trophic Transfer Assay

**Figure 3:**
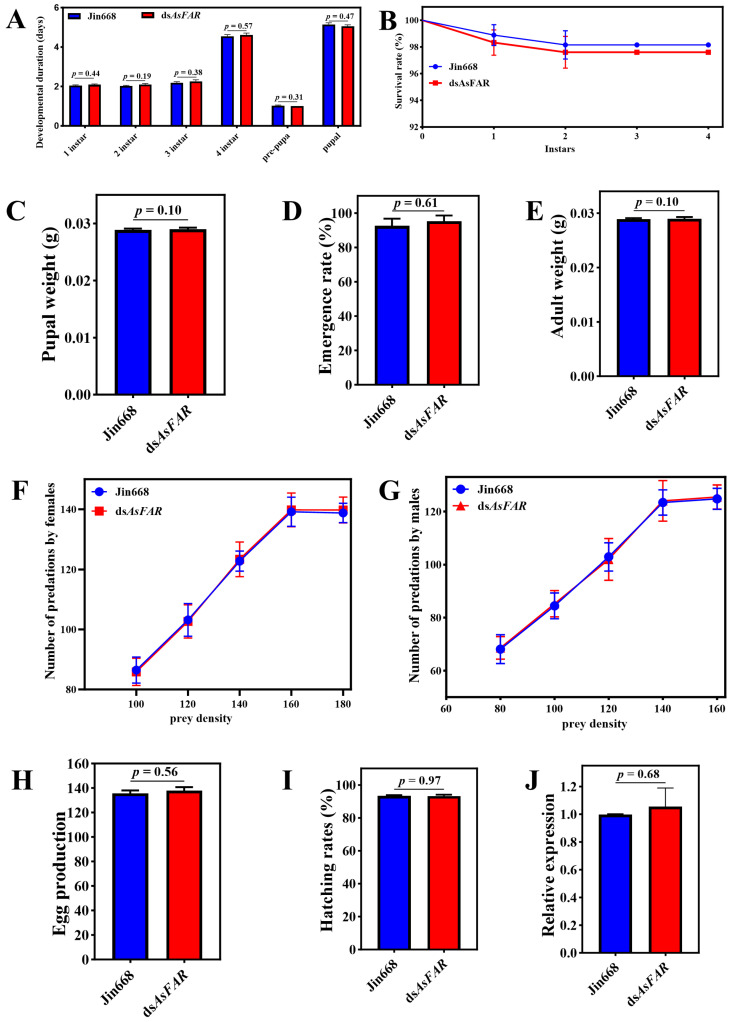
Life-table parameters of *H. axyridis* through the trophic transfer assay. Duration of the pre-imaginal stages (df_1 instar_ = 82, df_2 instar_ = 80, df_3 instar_ = 80, df_4 instar_ = 80, df_pre-pura_ = 80, df_pupal_ = 75) (**A**), survival rates (**B**), pupal weight (df = 80) (**C**), emergence rate (df = 80) (**D**), adult weight (df = 80) (**E**), number of predations by females (**F**), number of predations by males (**G**), egg production (df = 28) (**H**), and hatching rates (df = 28) (**I**) of *H. axyridis* after feeding on aphids that were reared on ds*AsFAR* and jin668 cottons (df = 28). (**J**) Changes in *FAR* expression after feeding on Jin668 and ds*AsFAR* cotton in *H. axyridis* (df = 4). The values are represented as mean ± SD of three replicates. Error bars indicate the standard error of the mean. Each group was compared by Student’s *t*-test.

The ds*AsFAR* transgenic cotton and its parent Jin668 were planted in the greenhouse, and the cotton aphids were transferred to different types of cotton plants for propagation and used to feed *H. axyridis*. The results showed that no significant difference was observed in the development duration (Figure 3A), survival rate (Figure 3B), pupal and adult weight (Figure 3C,E), number of predations by females (Figure 3F), number of predations by males (Figure 3G), egg production (Figure 3H), or hatching rate (Figure 3I) of *H. axyridis* when compared with the control treatment, indicating that there was no adverse effect of ds*AsFAR* transgenic cotton on the survival and development of *H. axyridis*. Adults of *H. axyridis* that fed on aphids reared on different types of cotton plants were collected and analyzed by qPCR. The results showed that the ds*AsFAR* transgenic cotton had no adverse effect on the homologous gene expression of *H. axyridis* through the trophic transfer assay (Figure 3J).

### 3.4. Off-Target Effects on Non-Homologous Genes Were Induced by dsAsFAR in H. axyridi

**Figure 4:**
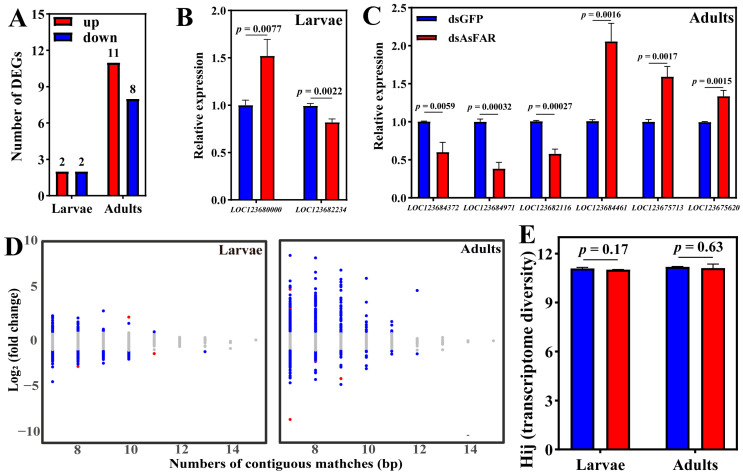
Transcriptome entropy for evaluating siRNA off-effects. Quantitative count of DEGs (**A**) for transcriptome sequencing and qRT-PCR validation for *O. similis* larvae (df = 4) (**B**) and adults (df = 4) (**C**). (**D**) The number of base pairs matched is linked with their fold changes for each continuous matched gene. Red, upregulated genes; blue, downregulated genes; gray, no significant genes. (**E**) The effect of siRNA feed on the Shannon transcriptome entropy (df = 4). The values are represented as mean ± SD of three replicates. Error bars indicate the standard error of the mean. Each group was compared by Student’s *t*-test.

According to the results of *H. axyridis* consumption of dsRNA, samples of *H. axyridis* that fed on ds*AsFAR* and ds*GFP* for 4 days at the larval and adult stages were sent to transcriptome sequencing. The results showed that exposure to ds*AsFAR* induced down-regulation of two genes in larvae and eight genes in adults, compared to ds*GFP*, but not in the *HaFAR* gene family (Figure 4A, Tables S2 and S3). We randomly selected eight differentially expressed genes (DEGs) for qRT-PCR assays to validate the transcriptome data. The expression profiles of all selected genes were consistent with the transcriptome results in both larvae and adults (Figure 4B,C). KEGG enrichment analysis of DEGs showed that DEGs in *dsAsFAR*-treated larvae were only significantly enriched in the non-homologous end-joining pathway; however, DEGs in adults were significantly enriched in protein processing in the endoplasmic reticulum, glycosaminoglycan degradation, neutrophil extracellular trap formation, and toxoplasmosis (Figure S1). The results of GO enrichment analysis were consistent with those of KEGG analysis, with both showing no correlation with lipid or wax synthesis (Figure S2). Therefore, we speculate that ds*AsFAR* may induce the off-target effects of the non-homologous genes in the *H. axyridis*.

### 3.5. The Evaluation of Off-Target Effects and Transcriptome Homeostasis

The interference fragment of ds*AsFAR* we selected did not find a homologous fragment in *H. axyridis* (E-value < 1 × 10^−5^). Through analysis of the genes containing continuous matches with dsRNA, the results showed that off-target effects were not correlated with the number of consecutive matching base pairs. Most DEGs of *H. axyridis* that fed on ds*AsFAR* have less than 10 consecutive matching base pairs. In addition, we also found that 99.90% to 100% of *H. axyridis* genes containing continuously matched regions with ds*AsFAR* were not affected (Table 1). The correlation between the number of matches and their fold changes showed that the highest expression changes were usually detected on genes with fewer consecutive matches (Figure 4D). This indicated that the causal relationships between the number of base matches and changes in their expression levels were diverse. Within the same gene function network, silencing of one gene may induce changes in the expressions of other genes in pathways that interact with level 1 genes (level 2 pathway in Table 2) and so on (level 3 pathway in Table 2).

Shannon entropy of a transcriptome reflects the overall variability in gene expression and can be used to assess potential risks to fundamental biological processes that may arise from exposure to exogenous dsRNA. The results showed that the Shannon entropy values did not change significantly following ingestion of ds*AsFAR* in both larvae and adults (Figure 4E), which demonstrates that ds*AsFAR* has no adverse effect on the transcriptome homeostasis of *H. axyridis*.

## 4. Discussion

Traditional chemical pesticides have historically played a crucial role in contemporary agriculture by effectively managing insect pests, weeds, and plant pathogens, thereby significantly enhancing crop yields and contributing to global food security. Nonetheless, their extensive application has generated substantial ecological and environmental concerns. Conventional chemical pesticides frequently display broad-spectrum toxicity, which can adversely affect non-target insects and plants. Additionally, these substances have the potential to leach into or run off into adjacent water bodies and soil, thereby amplifying their ecological impact [22].

In contrast, GM technology provides new ideas for increasing crop yields and improving the quality of agricultural products. With the widespread application of IRGE crops and other novel biotechnologies in insect-pest management [19], rather than synthetic insecticides, many concerns about both their negative and positive effects have arisen in many countries. An effective and standardized risk evaluation framework for genetically engineered crops is necessary before their promotion and application. Such a framework not only helps to prevent unintended consequences to public health, agriculture, and conservation, but also facilitates the authentication and communication of the same biotechnology product among governmental regulators worldwide. The more popular IRGE crops are transgenic Bt insect-resistant crops, for which many studies on their environmental risks have been published so far. RNAi technology, as an efficient tool for interpreting sequence function and expression, is widely used in various fields [23], especially in gene function analysis and genetic breeding of plants. For example, transgenic materials with inserted dsRNA have been used to increase tobacco’s resistance to insects [24] and to improve the resistance of cotton to cotton bollworm [25,26]. In addition, by integrating Bt toxin and RNAi, researchers have created transgenic cotton plants that target the juvenile hormone methyltransferase gene in cotton bollworm, effectively postponing insect pairing [27]. Additionally, RNAi has also been used to silence the potato beetle’s highly specific ecdysone-related receptor genes, resulting in a mortality rate up to 80% and a significant reduction in larval weight and pupal weight [28]. Although significant progress has been made in researching these new genetically modified materials, there are still many potential environmental safety risks, including gene drift and off-target effects. Importantly, the off-target problem of RNAi has gradually emerged as RNAi research has deepened [16]. Hence, the off-target effect of dsRNA has become one of the important indicators for the safety evaluation of transgenic dsRNA plants [29,30]. However, there is no conclusive evidence regarding their possible environmental risks.

Natural enemies of insects play a crucial role in agricultural ecosystems [31]. Within the plant–pest–natural enemy framework, previous assessments of the ecological risks associated with insect-resistant IRGE crops have predominantly focused on evaluating their potential effects on the development and survival of beneficial arthropods, especially through trophic transfer assays. For example, Chen et al. reported that miRNA-mediated transgenic rice targeting the stem borer had no significant effect on *Apis mellifera* Linnaeus (Hymenoptera: Apidae), an essential pollinator [32]. Toxicity evaluations based on trophic transfer within the food chain are comparable to the whole-food animal tests frequently utilized in regulatory assessments of the potential risks posed by IRGE crops to human health. Consequently, it is posited that food chain-based testing constitutes one of the most suitable approaches for assessing the exposure pathways affecting natural enemies within the plant–pest–natural enemy system. Nonetheless, alternative approaches and validation strategies should also be explored prior to its routine implementation. In parallel, animal feeding tests facilitate the administration of significantly elevated doses of specific insecticidal components, thereby incorporating a conservative or worst-case exposure scenario into the risk assessment framework. For instance, the U.S. Environmental Protection Agency recommends employing exposure levels that are 10 times or more than typical field concentrations in laboratory risk assessments [33]. This approach ensures a high degree of safety assurance. Nonetheless, the reliability of these assessments is contingent upon the availability of effective and nutritionally adequate artificial diets. Our study demonstrates that even under 10-fold (7 µg/g) high-dose exposure, ds*AsFAR* did not negatively impact the development and survival of *H. axyridis*, a key predator within the cotton plant–pest–natural enemy triad. Similarly, dsRNA targeting *Brassicogethes aeneus* Fabricius (Coleoptera: Nitidulidae) (*dsalphaCOP*) exhibited no adverse effects on the parasitoid *Nasonia vitripennis* Walker (Hymenoptera: Pteromalidae) [34]. Additionally, no toxic effects were observed in other beneficial arthropods, such as *Telenomus podisi* Ashmead (Hymenoptera: Scelionidae) [35], *Propylea japonica* Thunberg (Coleoptera: Coccinellidae) [36,37], *Cyrtorhinus lividipennis* Reuter (Hemiptera: Miridae) [38,39], *Ontsira mellipes* Ashmead (Hymenoptera: Braconidae) [40], and *Coleomegilla maculata* DeGeer (Coleoptera: Coccinellidae) [41]. Nonetheless, certain studies indicate that insecticidal dsRNA may present species-specific risks to natural enemies. For instance, ds*vATPase-A*, which targets *Diabrotica virgifera* LeConte (Coleoptera: Chrysometidae), has been shown to significantly, albeit marginally, extend the developmental duration of the predator *Adalia bipunctata* Linnaeus (Coleoptera: Coccinellidae) and to significantly decrease the survival rate of another predator, *Coccinella septempunctata* Linnaeus (Coleoptera: Coccinellidae) [42,43]. These findings highlight the importance of conducting species-specific safety assessments within the plant–pest–natural enemy interaction framework to ensure a comprehensive ecological risk evaluation for RNAi-based crops. However, one limitation of our study is that we did not assess the multigenerational or long-term effects on *H. axyridis*, which merits further investigation.

Bioinformatics is a powerful method for identifying off-target effects. Typically, most contemporary research employs bioinformatics during the initial phase of selecting and designing target genes for transgenic dsRNA crops [19]. Specialized software, dsCheck [44] and OfftargetFinder [45], are utilized to conduct off-target searches and to design dsRNA with minimal sequence similarity to non-target genes, thereby reducing the possibility of unintended off-target effects [46]. However, due to the limited availability of sequence information for all species, it is not feasible to predict off-target effects solely through bioinformatics. As scientific research progresses, omics analysis techniques can be employed from a comprehensive perspective to evaluate the off-target effects of RNAi [47]. Additionally, RNAi induces specific effects on the transcriptomes of organisms, which may pose risks due to its potential adverse impacts, such as off-target gene silencing, on non-target species in RNAi-modified crops [21]. These off-target effects in numerous insects have been evaluated using omics technologies [21,32,36]. Taning et al. [48] conducted in silico predictions and identified numerous potential off-target genes when targeting dsRNA for the pollen beetle pest. However, when they tested these candidates in in vivo experiments, no negative effects on bumblebees were observed [48]. In this study, our transcriptomic analysis also identified unintended DEGs that may be induced by ds*AsFAR* in larvae and adults of *H. axyridis*. However, a limitation of this study is that to ensure consistency and minimize technical variation, the same RNA samples were used for both RNA-seq and qRT-PCR validation. This approach may introduce circularity and does not fully account for biological variability. Whether these unintended DEGs are truly induced by the inference of *AsFAR*, and whether further validation with independent RNA samples would yield consistent results, warrants further investigation in future ecological risk assessment. Additionally, our findings indicated that off-target effects were not correlated with the number of consecutive matched base pairs. RNAi could be triggered with as few as seven continuous matched base pairs between the target gene and exogenous dsRNA, as demonstrated in our previous study [21]. This implies that transcriptomes exhibit a high degree of adaptability to external dsRNA, necessitating the consideration of unintended transcriptomic alterations in the safety assessments of RNAi crops for non-target organisms. Nonetheless, these alterations alone do not comprehensively encapsulate potential risks [21]. The application of Shannon entropy could facilitate the evaluation of transcriptome stability [49] and may serve as one of the effective approaches for ecological risk assessment.

## 5. Conclusions

Compared to classical synthetic pesticides, RNAi offers an innovative, specific, and environmentally sustainable pest control method. Nonetheless, there are multiple challenges to address at the technical, regulatory, and risk assessment levels [20]. In this study, we proposed a biological and ecological risk evaluation framework for RNAi crops on insects’ natural enemies, which includes assessing exposure routes for insecticidal dsRNA; testing food-chain delivery to reflect real-world conditions; conducting animal-feeding tests for worst-case effects; analyzing off-target effects for unintended silencing; and evaluating transcriptomic stability. In addition, our study also revealed that ds*AsFAR* did not exert any harmful impacts on *H. axyridis* at either the developmental or transcriptomic levels involving *FAR* or its related genes. In alignment with previous studies, our results highlight the importance of evaluating RNAi crops on an individual basis for their safety regarding non-target organisms, especially in relation to off-target consequences. Further investigation is necessary to understand the mechanisms involved. We hope this study will support the development of guidelines for biological and ecological risk assessment to minimize the potential negative consequences of RNAi crops under a plant–pest–natural enemy ecological interaction framework.

## Figures and Tables

**Table 1:** The silencing of the genes containing continuously matched regions with ds*AsFAR* in the *H*. *axyridis* transcriptome.

No. of Matched Base Pairs (bp)	Total No. of Genes	No. and Percentage of Up-Regulated Genes	No. and Percentage of Down-Regulated Genes	No. and Percentage of Non-Significant-Change Genes
7	5462	3 (0.05%)	3 (0.05%)	5456 (99.90%)
8	3722	2 (0.05%)	1 (0.03%)	3719 (99.92%)
9	1272	0 (0)	1 (0.08%)	1271 (99.92%)
10	325	0 (0)	0 (0)	325 (100%)
11	103	0 (0)	0 (0)	103 (100%)
12	21	0 (0)	0 (0)	21 (100%)
13	9	0 (0)	0 (0)	9 (100%)
14	3	0 (0)	0 (0)	3 (100%)
15	1	0 (0)	0 (0)	1 (100%)
Total	10,918	5 (0.05%)	5 (0.05%)	10,908 (99.90%)

**Table 2:** The silencing of the related KEGG genes after ds*AsAFR* ingestions in *H. axyridis*.

Transcriptome	KEGG Pathway Level	Total No. of Genes	No. and Percentage of Up-Regulated Genes	No. and Percentage of Down-Regulated Genes	No. and Percentage of Non-Significant Change Genes
	Level 2	2595	0 (0.00%)	0 (0.00%)	2595 (100.00%)
Larvae	Level 3	23,952	7 (0.03%)	3 (0.01%)	23,942 (99.96%)
	Total	26,547	7 (0.03%)	3 (0.01%)	26,537 (99.96%)
	Level 2	2698	190 (7.04%)	6 (0.22%)	2502 (92.74%)
Adults	Level 3	25,545	1261 (4.94%)	39 (0.25%)	24,245 (94.91%)
	Total	28,243	1451 (5.14%)	45 (0.16%)	26,747 (94.70%)

## Data Availability

The raw sequence data are available in the China National Center for Bioinformation (https://ngdc.cncb.ac.cn/) under accession number PRJCA043540.

## Supplementary Materials

The following supporting information can be downloaded at https://www.mdpi.com/article/10.3390/biology14091173/s1, Figures S1 and S2: The KEGG analysis of transcriptomes and the GO analysis of transcriptomes, respectively; Tables S1–S3: Primers used for this study, DEGs in *H. axyridis* adults fed ds*GFP* vs. ds*AsFAR*, and DEGs in *H. axyridis* larvae fed ds*GFP* vs. ds*AsFAR*, respectively.
