# Supplementary material for: Assessment of Potential Toxic Effects of RNAi-Based Transgenic Cotton on the Non-Target Predator Harmonia axyridis
Source: Biology (Basel). 2025 Sep 2;14(9):1173. doi: 10.3390/biology14091173 (PMC12467772; doi:10.3390/biology14091173)
Supplement: Supplementary file 1 [file biology-14-01173-s001.zip › biology-3746770-supplementary.pdf]

Supplementary Materials

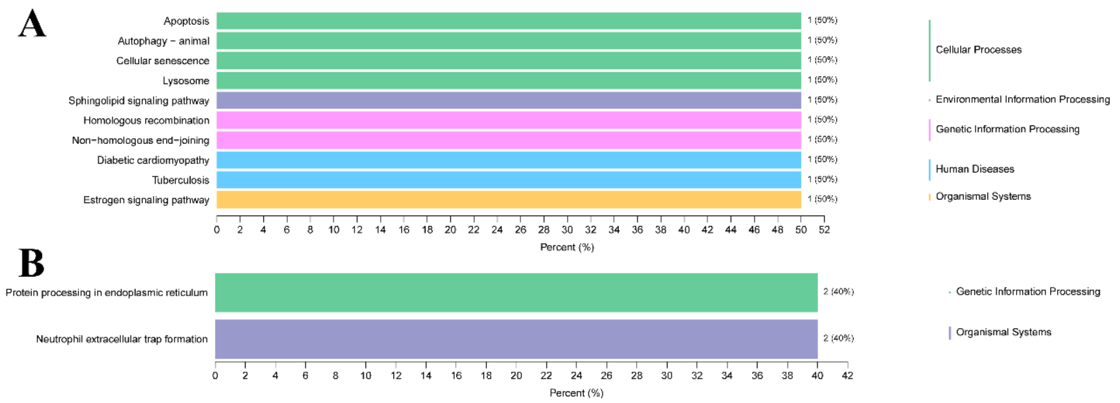

Figure S1. The KEGG analysis of transcriptomes.

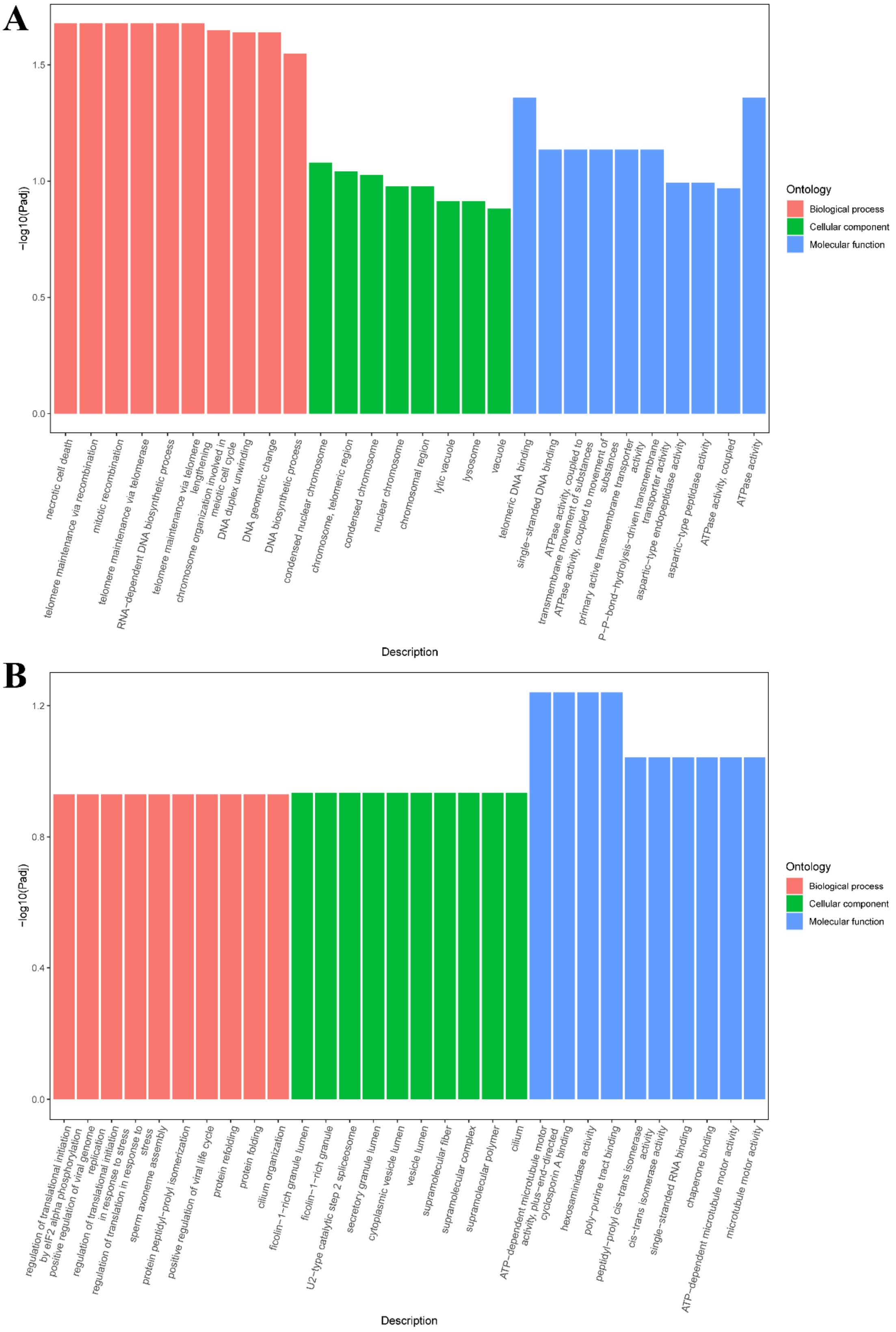

Figure S2. The GO analysis of transcriptomes.

Table S1. Primers used for this study

Primer Name	Primer sequence (5’ - 3’)
ds*Asfar*-F	taatacgactcactatagg GCGAATGGTTGAAAGAAAATAGG
ds*Asfar*-R	taatacgactcactataggGTTGGTGAAAGTGTAGGTGTTGG
ds*Hafar*-F	taatacgactcactataggGGCTGCAAGAGAACCCAAAAG
ds*Hafar*-R	taatacgactcactataggGTGATGGCTTCTCTCCAATCA
q*Hafar*-F	TAATGGACCTGTAGGACTG
q*Hafar*-R	CCTGATTTCTAATGCTGGA
q*18S*-F	ACGGACTTCGGTAGGACG
q*18S*-R	CGCAGACAATCCCGAAA

Table S2. DEGs in *H. axyridis* adults fed ds*GFP* vs. ds*AsFAR*

ID	log2FoldChange	pvalue	padj	regulated	chr
LOC123684372	-6.51678139	4.61E-12	4.88E-08	down	NC_059501.1
LOC123675654	5.863211429	5.99E-08	0.000317	up	NC_059503.1
LOC123682912	3.459850233	1.1E-07	0.000387	up	NC_059506.1
LOC123684971	-2.795162486	1.41E-06	0.003725	down	NC_059507.1
LOC123675790	-8.484486222	2.23E-06	0.00424	down	NC_059503.1
LOC123681926	-7.101185322	2.76E-06	0.00424	down	NC_059506.1
LOC123678048	9.872347697	2.8E-06	0.00424	up	NC_059504.1
LOC123684461	4.867282141	4.38E-06	0.005794	up	NC_059507.1
LOC123682116	-3.346188297	8.24E-06	0.009698	down	NC_059506.1
LOC123675713	2.075279713	1.55E-05	0.015806	up	NC_059503.1
LOC123675620	2.267659916	1.68E-05	0.015806	up	NC_059503.1
LOC123674110	5.662738993	1.92E-05	0.015806	up	NC_059502.1
LOC123683319	-1.044608573	1.94E-05	0.015806	down	NC_059506.1
novel.8	7.945178199	2.26E-05	0.017076	up	NC_059501.1
novel.771	6.806997465	4.82E-05	0.031344	up	NC_059502.1
LOC123681925	-4.043012832	4.83E-05	0.031344	down	NC_059506.1
LOC123674081	-2.118041382	6.15E-05	0.034252	down	NC_059501.1
LOC123672031	3.394830575	7.61E-05	0.040261	up	NC_059502.1
LOC123678217	5.735694097	8.15E-05	0.041079	up	NC_059504.1

Table S3. DEGs in *H. axyridis* larvae fed ds*GFP* vs. ds*AsFAR*

ID	log2FoldChange	pvalue	padj	regulated	chr
LOC123683033	1.271390239	1.02723E-06	0.005478388	up	NC_059501.1
LOC123680000	2.649740578	1.06139E-06	0.005478388	up	NC_059505.1
LOC123675973	-2.722813641	1.07662E-05	0.027784893	down	NC_059503.1
LOC123682234	-1.326839949	3.11699E-05	0.045966661	down	NC_059506.1
